# Neuroimmune Mechanisms in Traumatic Brain Injury and Cancer: Parallel Courses or Existence in Different Orbits

**DOI:** 10.3390/biomedicines14010112

**Published:** 2026-01-06

**Authors:** Mariia Zhukova, Natalia Ermakova, Edgar Pan, Evgenii Skurikhin

**Affiliations:** Institute of General Pathology and Pathophysiology, 125315 Moscow, Russia; nejela@mail.ru (N.E.); artifexpan@gmail.com (E.P.); eskurihin@inbox.ru (E.S.)

**Keywords:** traumatic brain injury, cancer, systemic inflammation, neuroinflammation, autonomic nervous system, T-cell exhaustion

## Abstract

Traumatic brain injury (TBI) and malignancies, despite their distinct nature, are characterized by similar immune responses, including the development of local and systemic inflammation and T-cell exhaustion. This article compares the role of immune checkpoints in the development of immune dysfunction in cancer and TBI, examines the contribution of the sympathetic nervous system to these changes, and discusses the relationship between local and systemic inflammation in these two conditions. Particular attention is paid to approaches to pharmacological modulation of inflammation and the impact on exhausted T-cells in these conditions. Comparison of inflammation and T-cell exhaustion in cancer and TBI highlights existing gaps in our understanding of immune regulation in TBI and points to areas requiring further investigation. Clarification of the immune mechanisms underlying the pathogenesis of TBI may facilitate the search for new diagnostic markers and lay the groundwork for the development of new therapeutic approaches for TBI treatment.

## 1. Introduction

Inflammation is a fundamental process, serving as the body’s first line of defense against various damaging factors. It is important to note that inflammation has a dual nature. While acute inflammation is aimed at localizing the pathological process, its rapid resolution, and the restoration of damaged tissue, chronic inflammation is an integral component of several diseases [1]. Chronic inflammation is a risk factor for the development of cancer, autoimmune diseases (systemic lupus erythematosus, rheumatoid arthritis), metabolic disorders (obesity, diabetes mellitus), atherosclerosis, lung (bronchial asthma, chronic obstructive pulmonary disease), skin (psoriasis), and intestines (Crohn’s disease, ulcerative colitis) diseases [2,3]. Local inflammation promotes cancer metastasis and progression [4].

In addition, prolonged antigenic stimulation that occurs during chronic inflammation, humoral and cellular inflammatory components, creates conditions for T-cell exhaustion This phenomenon was first observed in viral infections and was later described in chronic non-infectious diseases. Briefly, exhaustion is a specific condition in which T-cell effector function is reduced. Currently, various independent research groups are focusing on T-cell exhaustion in cancer and its contribution to treatment response and disease outcome [5,6].

T-cell exhaustion is not limited to cancer. For example, dysfunctional T-cells have been detected in the synovium of patients with rheumatoid arthritis. In patients with systemic lupus erythematosus, T-cell expression of the ‘exhaustion markers’ such as PD-1, CD57, and EOMES was higher than in healthy volunteers. Exhausted CD39^+^ T-cells have been observed in patients with Crohn’s disease [7,8]. An immunophenotype characteristic of exhaustion was recorded in T-cells of patients with latent autoimmune diabetes in adults [9].

Given the existing link between the immune system and the central nervous system, the development of T-cell exhaustion in neuroinflammation is of particular interest. Neuroinflammation is one of the key factors in neurodegeneration in Alzheimer’s disease, amyotrophic lateral sclerosis, Huntington’s disease, and Parkinson’s disease [10,11]. Factors causing neuroinflammation include genetic predisposition, old age, systemic inflammation, the presence of foci of chronic inflammation, etc. [10]. The role of traumatic brain injury (TBI) in the development of neuroinflammation and cell exhaustion is of particular interest. According to statistics, more than 20 million cases of TBI were registered in 2021, and it is projected to remain a leading cause of disability and mortality through 2030 [12,13]. Improving the effectiveness of patient treatment is possible through the development of new therapeutic strategies. However, this requires an understanding of the underlying mechanisms of traumatic brain injury. In particular, it remains unclear whether the mechanisms of T-cell exhaustion in cancer and TBI are similar or unique to each pathology.

In this review, we attempted to summarize the available data on the relationship between local and systemic inflammation, with a focus on inflammation in TBI. We also analyzed the available data on T-cell exhaustion in TBI compared to cancer, where this phenomenon has been best studied. We also provide an overview of methods for correcting neuroinflammation and a list of agents with the potential to reduce it.

## 2. The Immune System Alteration

### 2.1. The Immune System in Traumatic Brain Injury

TBI is a pressing issue in modern healthcare. In 2019 alone, more than 27 million new cases of TBI were registered, and approximately 7 million cases of related disability were reported [14]. According to current concepts, TBI damages brain neurons, leading to neuroinflammation and white matter demyelination. Chronic neuroinflammation, with activation of microglia and astrocytes, raises the risk of neurodegenerative processes and contributes to their further progression, increasing the risk of Alzheimer’s disease and multiple sclerosis [15]. In this regard, the immune system in TBI is a significant object of research, since there are still no effective strategies to combat such a pathological component of the disease as neuroinflammation.

It is known that TBI is accompanied by significant changes in leukocyte and cytokine profiles in peripheral blood. However, the data presented in the literature vary significantly. This is explained by the time frame of observation, different approaches to injury modeling, and the subject and design of the study. For example, TBI has been shown to increase the number of certain leukocyte subpopulations in the blood of patients, simultaneously with a change in their functional activity [16,17]. A study of patients with TBI showed leukocytosis and neutrophilia 24 h after injury. At the same time, neutrophil phagocytic activity in patients was lower than in healthy volunteers. The authors note that under these conditions, serum concentrations of TNF-α, IL-6, and C-reactive protein (CRP) were elevated for up to two weeks of observation [16]. Such ambiguous data on the systemic immune response make it difficult to study underlying mechanisms of traumatic brain injury.

TBI is often accompanied by immunosuppression. Schwulst S.J. et al. (2013) [18] showed a decrease in the level of pro-inflammatory cytokines (IL-12, INF-γ, CCL5) in the blood serum (by day 14) and thymus weight (on days 3 and 60) in mice with TBI. A study of the mononuclear phagocyte system revealed a deficiency of monocytes in the blood (day 30) and macrophages in the spleen (day 60). A detailed analysis of macrophage subpopulations in the spleen of mice with brain injury showed a decrease in the number of pro-inflammatory M1 macrophages, while the number of anti-inflammatory M2 macrophages, on the contrary, increases.

In addition to changes in the overall leukocyte count, individual cell subpopulations also undergo changes. The number of NK-cells tends to decrease in TBI. A significant decrease in the number of CD3^−^CD56^+^ cells was observed in the peripheral blood of patients who had sustained TBI compared to healthy volunteers. The degree of cell reduction correlated with the severity of the injury [19]. NK-cells demonstrated decreased expression of the activation markers CD69 and CD25 and reduced IFN-γ production [20].

Data on the role of NKT-cells in TBI are limited. There is evidence that the number of circulating NKT-cells decreased 4 days after injury, as did the level of perforin in the cells [21]. However, in other conditions, such as ischemic stroke and neurodegenerative diseases, an increase in the number of NKT-cells in the blood and their infiltration into the brain have been observed. However, their role in the pathogenesis of CNS diseases is controversial and requires further study [22].

The γδ T-cell subpopulation increased during the first day after experimental TBI [23]. The potential for pro- or anti-inflammatory activity depended on the structure of the T-cell receptor γ chain. Vγ1 γδ T-cells secrete TGFβ, reducing microglia activation and attenuating neuroinflammation. Vγ4 γδ T-cells, on the contrary, activate microglia and enhance neuroinflammation through the production of IL-17 and IFNγ [24].

The role of B-cells in TBI is currently poorly understood. The frequency of circulating B-cell and IL-10^+^ B-cells was higher after brain injury due to trauma or stroke compared to healthy volunteers [25]. The protective effect of B lymphocytes in TBI is associated with an increase in IL-10, TGFβ, IL-35, and a decrease in the production of TNFα, INF-γ, and IL-6 by myeloid cells such as monocytes/macrophages [26].

Mechanisms of immunosuppression in TBI include the release of damage-associated molecular patterns (DAMPs), an increase in myeloid-derived suppressor cells (MDSCs), and altered autonomic nervous system function [27]. The initiating moment in the development of immune dysfunction following TBI is believed to be the release of DAMPs such as glial protein S100B, HMGB1, and ATP [28]. In response, microglia produce several cytokines, including IL-1β and IL-6, which enter the bloodstream and promote the recruitment of immune cells, including MDSCs, from the periphery to the brain [29,30]. Additionally, HMGB1 promotes MDSC expansion via the receptor for advanced glycation end-products [31]. It is believed that the immunosuppressive activity of MDSCs in TBI is associated with:

- Altered L-arginine metabolism and competition between MDSCs and T-cells.
- Production of reactive oxygen and nitrogen species, which leads to nitration of TCR and CD8 receptor and disrupts MHC-mediated T-cell stimulation [32]. These mechanisms are largely similar to the processes occurring in cancer.

MDSC subpopulations differ in their functional activity. G-MDSCs more actively produce reactive oxygen species, while M-MDSCs produce more nitric oxide. By expressing more iNOS, M-MDSCs more actively suppress immune cell function by inhibiting T-cell JAK3 and STAT5 [33].

The contribution of MDSC subpopulations to the development of dysfunctional changes in the immune system is discussed. Experiments have shown the accumulation of CD45^hi^CD11b^+^Ly6C^+^ MDSCs in the brain during the first day after experimental injury, with increased expression of TNFα, IL-1β, and TGFβ by CD45^hi^CD11b^+^ cells [34]. Another study showed that the number of G-MDSCs (Ly6C^lo^Ly6G^+^) gradually decreased from day 1 to day 7 in the mouse brain after controlled cortical impact. On the contrary, the number of M-MDSCs (Ly6ChiLy6G^−^) gradually increased during the same observation periods. All this occurred against the background of a gradual decrease in the total MDSC population, defined as CD11b^+^Gr-1^+^ cells. Ex vivo, CD11b^+^Gr-1^+^ cells suppressed CD4^+^ cell proliferation [35].

Using a spinal cord injury (contusion injury) model, Saiwai H et al. (2013) [36] showed that depletion of Ly6C^+^Ly6G^−^ cells infiltrating the spinal cord was associated with increased recovery time for hindlimb function in mice after injury. Transplantation of Ly6C^+^Ly6G^−^ cells to injured animals was accompanied by increased levels of anti-inflammatory IL-10 and TGF-β, as well as increased levels of VEGF, IGF, and HGF, which can exert anti-inflammatory, neuroprotective, and anti-apoptotic effects.

### 2.2. The Immune System in Cancer

The development of non-hematological malignancies is often characterized by leukocytosis, which is considered a manifestation of paraneoplastic syndrome. Growth factors, such as G-CSF and GM-CSF, secreted by the tumor stimulate the proliferation and differentiation of hematopoietic precursors, inducing granulocytopoiesis in the bone marrow. Some cytokines (IL-1α, β, IL-3, and IL-6) also contribute to the development of leukocytosis [37]. An increase in the C-C motif chemokine ligand 2 (CCL2) blood concentration, observed in patients with solid tumors, induces the mobilization of macrophages from the bone marrow [38].

In addition, changes in individual subpopulations of immune cells are observed. In cancer patients, NKT-cell depletion in the blood and tissues was detected [39,40], proliferative activity and the ability to produce INF-γ cytokines decreased [39,41]. A decrease in the NKT-cell number, as well as their functional activity, did not depend on the tumor type [42].

Peripheral blood NK-cells demonstrated a decrease in the production of IFN-γ and TNF-α, and a reduction in the expression of activating molecules NKp30, NKG2D, DNAM-1, and CD16 [43]. However, no significant change in their numbers was detected [44].

γδ T-cells contribute to the formation of the immunosuppressive tumor microenvironment by secreting IL-10, TGF-β, adenosine and by expressing PD-L1 and galectin-9 [45]. The Vδ2 subpopulation of γδ T-cells is additionally capable of suppressing the proliferation of CD4^+^ αβ T-cells and their IL-2 production [46]. IL-17-producing γδ T-cells promote the expansion of neutrophils into the tumor and, as a consequence, the suppression of CD8^+^ cell function and an increase in tumor metastatic activity [47].

Information about B-cell changes in hematological malignancies is extremely scarce. They mainly focus on their prognostic value [48,49,50]. When assessing the functional activity of B-cells, attention is focused on their potential pro-tumor properties. B-cells are able to suppress the immune response by secreting IL-10, IL-35, and TGF-β, stimulating the formation of regulatory T-cells (Treg) [51].

While the role of DAMPs and MDSCs in TBI remains to be clarified, their contribution to the development of an immunosuppressive microenvironment in malignancies is well-studied. MDSCs compete with immune cells for amino acids such as arginine, cysteine, and tryptophan, which are necessary for the activation and proliferation of T-cells. Moreover, MDSCs initiate an increase in the concentration of extracellular adenosine, a product of the enzymatic degradation of ATP, which inhibits CD3/CD28 T-cell activation. By disrupting the phosphorylation of Zap70, ERK, and Akt, MDSCs negatively affect the priming of lymphocytes mediated by antigen-presenting cells (endothelial cells of the liver sinusoids, splenic dendritic cells) [52,53]. In addition, MDSCs are able to induce INF-γ- and IL-10-dependent formation of Tregs, which are able to inhibit the activation and proliferation of cytotoxic T-cells [54]. Some of the MDSC subtypes activate Fas-mediated T-cell apoptosis and induce M2 macrophage polarization [55,56]. Additionally, expression of immune checkpoints (PD-L1) and formation of reactive oxygen and nitrogen species by MDSCs contribute to immunosuppression [53].

G-MDSCs are believed to be more active producers of reactive oxygen species and peroxides. M-MDSCs, in turn, are sources of reactive nitrogen species and arginase 1. Furthermore, M-MDSCs secrete C-C chemokine receptor type 5 (CCR5), which promotes tumor infiltration by Treg [57]. According to some data, G-MDSCs exhibit less pronounced immunosuppressive properties against T-cells (on a per-cell basis) due to the short life-time of peroxides and the need for close contact with the target cell to exert the effect [58].

DAMPs, including the S100 proteins, amphotericin (HMGB1), nucleic acids, and other components released from destroyed or damaged tumor cells, are capable of activating toll-like receptors (TLRs) on suppressor cells. Furthermore, some DAMPs, such as HMGB1 and PAUF, promote the differentiation and enhancement of functional activity of MDSCs [59,60]. Adenosine reduces T-cell and NK-cell activity and stimulates the expansion of Tregs by activating A2A receptors. S100 promotes the accumulation of MDSCs, whose immunosuppressive role was described above [61].

Thus, similar mechanisms of immunosuppression, including the MDSCs and the DAMPs release, have been identified in TBI and cancer. These common targets may form the basis for new approaches to correcting immunosuppression in these two diseases.

## 3. Immune Checkpoints

Immune checkpoints are surface proteins on the immune cells that regulate the immune response. Normally, they prevent excessive immune activity, preventing the development of autoimmune processes. The role of immune checkpoints in cancer has been best studied. Cytotoxic T-lymphocyte-associated protein 4 (CTLA-4), expressed by CD4^+^ and CD8^+^ T-cells, is highly homologous to the co-stimulatory molecule CD28. However, CTLA-4 has a higher affinity for the ligands B7-1 (CD80) and B7-2 (CD86) compared to CD28. This explains why CTLA-4 inhibits co-stimulatory signaling through CD28 [62]. In addition, CD80 and CD86, after binding to CTLA-4, are capable of undergoing endocytosis and subsequent degradation [63].

Lymphocyte-activation gene-3 (LAG-3, CD223) is an immunoglobulin family membrane protein, an immune checkpoint expressed on activated T-, B-, NK-cells, and dendritic cells. 73% of CD4^+^ T-cells and 76% of CD8^+^ T-cells isolated from biopsy specimens of patients with follicular lymphoma co-expressed PD-1 and LAG-3. This was associated with a significant decrease in T-cell function, unfavorable histological pattern, and poor survival. LAG-3 checkpoint blockade, in turn, increased the cytotoxic activity of T-cells [64].

PD-1 is well known as a marker of T-cell exhaustion in cancer [65]. PD-1 overexpression on T-cells is maintained by prolonged TCR stimulation, under the influence of some cytokines (IFN-γ, TGF-β, VEGF-A, IL-2, IL-6, IL-7, IL-12, IL-15, and IL-21), hypoxia, and other factors [66]. The interaction of PD-1 with its ligands PD-L1/PD-L2 induces phosphorylation of ITIM/ITSM, which suppresses PI3K/AKT and RAS signaling pathways, promoting the development of immune cell exhaustion [67]. Bengsch B. et al. (2016) [68] demonstrated the role of the PD-1/PD-L1 signaling pathway in reducing glycolysis and oxidative phosphorylation, as well as in the depolarization of mitochondrial membranes and an increase in the reactive oxygen species production by T-cells exhausted as a result of infection of animals with the lymphocytic choriomeningitis virus (strain Cl-13).

On the other hand, a study by Odorizzi P.M. et al. (2015) showed that prolonged antigen stimulation contributed to T-cell exhaustion in PD-1-deficient mice, namely, a decrease in IFN-γ production, an increase in the expression of LAG-3, CD244, CD160, and TIGIT genes, as well as a decrease in cell survival and proliferative activity [69]. Thus, PD-1 expression is not an unambiguous condition for T-cell exhaustion.

Soluble forms of PD-1 and PD-L1 (sPD-1 and sPD-L1, respectively) are considered as potential biomarkers and predictors of cancer progression [70,71,72]. As in non-small-cell lung cancer (NSCLC), high sPD-L1 level was associated with lower survival and progression-free survival. In cervical cancer, sPD-1 concentration was significantly higher in patients with stages II-IV disease. The sPD-L1 content in peripheral blood samples of patients with stage IV was significantly increased compared to patients with stages I-III [72].

In contrast to the large body of data on immune checkpoints in cancer, information on the role of checkpoints in TBI is sparse. Liu et al. (2024) showed that sPD-1 and sPD-L1 levels were significantly elevated in the blood of patients with severe TBI and pneumonia, which, according to the authors, may serve as a predictor of infectious complications [73]. The study by Yang Yi et al. (2019) also showed an increase in the number of PD-1-positive T-cells in the peripheral blood of TBI rats [74]. Interestingly, the percentage of PD-1-expressing T-cells increased in animals with spinal cord injury [75].

In TBI, the PD-1/PD-L1 signaling pathway likely plays a protective role, preventing the development of excessive inflammation after injury. Chen Q et al. (2019) [76] showed that PD-L1 expression on microglia, the brain’s resident macrophages, increased significantly after surgical brain injury. Moreover, changes in PD-L1 expression by microglia correlated with the level of its activation. PD-L1 blockade with monoclonal antibodies, in turn, increased cerebral edema in areas surrounding the injury, leading to activation of microglia and astroglia in vivo. The concentration of pro-inflammatory cytokines, such as IL-6 and iNOS, in the peripheral blood of animals receiving anti-PD-L1 antibodies was higher compared to control animals.

PD-1/PD-L1 blockade in experimental autoimmune encephalomyelitis was accompanied by an increase in the number of pro-inflammatory T-cells (Th1 and Th17), the concentration of pro-inflammatory cytokines and their production by microglia, and a decrease in the production of anti-inflammatory IL-10. PD-L1-positive astrocytes reduced the activity of PD-1-positive microglia in a model of autoimmune encephalitis. All of this suggests a role for PD-L1 in the resolution of autoimmune inflammation [77].

## 4. The Sympathetic Nervous System in Immune Regulation

Numerous reviews have presented in some detail the relationship between the immune system and the sympathetic nervous system (SNS) [78]. β2-adrenergic receptors are expressed on T-cells, dendritic cells, macrophages, and mediate sympathetic influence [79,80]. The role of the SNS in regulating immune system function is controversial. For example, stimulation of adrenergic receptors can simultaneously activate priming in lymph nodes and suppress immune cell-mediated inflammation in peripheral tissues, one mechanism of which may be a decrease in the production of TNF-α and IL-12 by immune cells [81,82]. On the other hand, SNS activation triggers myelopoiesis and induces immune cell mobilization from the bone marrow into the bloodstream [83].

### 4.1. Cancer

The SNS influences tumor growth and metastasis. Clinical trials show that high activity of this component of the nervous system is an unfavorable prognostic factor in prostate cancer, breast cancer, gastric cancer, lung cancer, and liver tumors [84,85,86,87,88].

The mechanisms by which SNS stimulates tumor growth include increased expression of immune checkpoints by T-cells, disruption of TCR signaling, antigen presentation by dendritic cells, and induction of Tregs formation [89,90,91].

### 4.2. Traumatic Brain Injury

**Figure 1:**
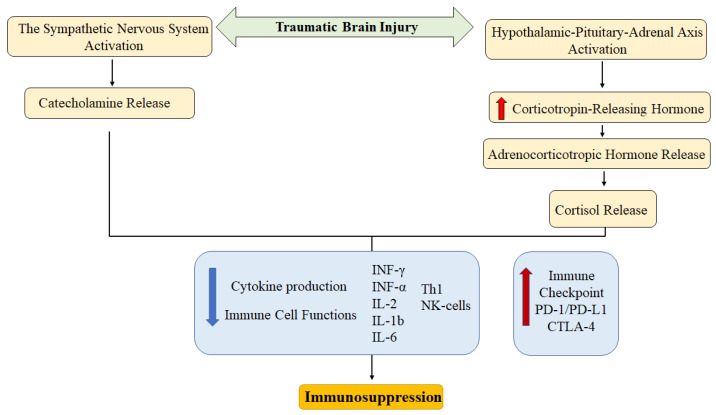
Basic neuroimmune mechanisms of immunosuppression after traumatic brain injury mediated by the sympathetic nervous system and the hypothalamic–pituitary–adrenal axis [29]. The red arrow indicates upregulation (increase in expression or levels), and the blue arrow indicates downregulation (decrease in expression or levels).

Data on the influence of the sympathetic nervous system on the development of TBI are limited. Yang Y et al. (2019) [74] linked the CD4^+^ and CD8^+^ T-cell exhaustion in TBI with SNS activity. In the experiment, it was shown that propranolol can successfully combat TBI-induced PD-1 expression on CD4^+^ and CD8^+^ T-cells in Sprague-Dawley rats. PD-1 expression on T-cell was reproduced by norepinephrine in vitro. These data indicate a link between the SNS and the PD-1/PDL-1 signaling pathway in TBI. The main mechanisms of immunosuppression after TBI associated with the neuroimmune axis are shown in Figure 1.

## 5. Interaction Between Local and Systemic Inflammation

### 5.1. Cancer

Inflammation is a physiological reaction that develops in response to physical or chemical factors, or the introduction of a pathogen. It is an important component of the immune response, necessary for pathogen elimination and tissue repair [92,93]. However, inflammation contributes to the development of chronic non-communicable diseases. For example, local and systemic inflammation is characteristic of chronic obstructive pulmonary disease and systemic connective tissue diseases [94,95].

The role of systemic and local inflammation in cancer is paying close attention. While acute inflammation can enhance treatment response, chronic inflammation, conversely, is associated with lower treatment efficacy and a high risk of metastasis and recurrence. Chronic local inflammation is a component of the tumor microenvironment, maintaining genomic instability and promoting metabolic reprogramming of tumor cells. Some authors identify TNF-α, IL-6, IL-1β, and IL-23 as key cytokines in this process [93].

A high level of local inflammation in cancer, typically measured by the number of tumor-infiltrating T-cells (TIL), is considered a marker of a positive prognosis. So-called immune-inflamed or “hot” tumors are characterized by their intense TIL infiltration, a high mutational load, and greater sensitivity to immune checkpoint inhibitors [96]. Accumulating clinical data indicate that the number of tumor-infiltrating CD8^+^, CD3^+^, and CD4^+^ T-cells (TIL) is positively correlated with longer recurrence-free and overall survival [97,98].

The local inflammatory response in cancer can, in turn, induce a systemic inflammatory response. This occurs due to the release of cytokines (IL-6, TNF-α) and exosomes secreted by the tumor into the bloodstream. This, like leukocytosis, is often considered part of the paraneoplastic syndrome [99].

The development of systemic inflammation in cancer is associated with a poor prognosis. Clinical trials showed that higher levels of systemic inflammation markers, such as the neutrophil-to-lymphocyte ratio (NLR) and the systemic immune inflammation index (SII), correlate with poorer survival rates and larger tumor size [100,101,102,103,104]. There is evidence of a link between C-reactive protein (CRP) levels and survival rates in patients with pancreatic and colorectal cancer [105,106,107,108].

### 5.2. Traumatic Brain Injury

The blood–brain barrier (BBB) is an obstacle for immune system cells, making the brain immunologically privileged [109]. For this reason, migration of inflammatory cells to the injury site becomes possible in the presence of BBB dysfunction or disruption, which is observed in TBI. Structural and functional changes in the BBB are observed both in the acute phase and in the late stages, and can persist for years. This contributes to the development of long-term neurological outcome [110,111].

Inflammatory cells in TBI may originate from the peripheral blood. Cerebral hypoperfusion and increased expression of ICAM-1 and E-selectin by endothelial cells facilitate the recruitment of immune cells to the damaged brain. Having migrated to the brain, T-cells interact with MCH I and MCH II, which triggers the processes of their proliferation and differentiation [82].

In response to injury, granzyme B production by T-cells increases, which can induce neuronal apoptosis [112]. The content of CD8^+^ T-cells expressing granzyme B increased significantly in the injured mouse brain at 8 and 32 weeks post-TBI [113]. Granzyme B-secreting T-cells shift the balance between Th2 and Th17 cells towards the latter, thereby aggravating demyelination in chronic inflammation [113]. Th1 and Th17 T-cells secrete INF-γ, TNF-α, and IL-17, promoting proinflammatory polarization of microglia [114,115].

Neutrophils migrating into the brain secrete matrix metalloproteinases 9 (MMP9) and 13 (MMP13), thus contributing to the development and maintenance of neuroinflammation after injury [116]. Together with reactive oxygen and nitrogen species, MMPs are capable of aggravating the disruption of the BBB [117]. Neutrophil extracellular traps (NETs) may contribute to cerebral hypoperfusion and thus exacerbate neurological deficits in TBI [118]. NET formation correlated with the severity of intracranial hypertension and the severity of neurological impairment in patients with TBI [119].

The humoral immunity also apparently contributes to the development of neuroinflammation. In vivo experiments have shown the appearance of autoantibodies in the blood after TBI [113]. Although the concentration of such “brain” antigens as glial fibrillary acidic protein (GFAP), myelin basic protein (MBP), neuron-specific enolase (NSE), glial protein S100B, ubiquitin carboxy hydrolase L1 (UCHL1), and neurofilament proteins decreases in peripheral blood several weeks after TBI, antibodies to them were detected for a long time after the injury [82].

Another mechanism for the development of neuroinflammation in TBI is the release of DAMPs, which lead to the activation of microglia and increased production of a whole spectrum of pro-inflammatory cytokines and chemokines by microglia: IL-1β, IL-6, IL-12, TNF-α, CCL2, and CXCL9. Microglial cells are essentially resident macrophages and are considered the main cells involved in the initiation and maintenance of neuroinflammation in brain damage of various origins (trauma, stroke, neurodegenerative diseases) [120,121]. These cells are capable of acquiring the functional characteristics of both phagocytes and antigen-presenting cells [122]. In the acute phase, this promotes the elimination of damaged tissue, stimulates reparative processes, and provides neuroprotection [123].

Following injury, microglial cells, which are resident macrophages, polarize toward M2 and acquire an activated immunophenotype that can persist for an extended period. Experimental studies have shown that markers of activated MCH II^+^ microglia were detected months after TBI in various brain regions (the cerebral cortex, thalamus, striatum, and corpus callosum) [124].

Cytokines appear to be the primary link between the central nervous system (CNS) and the immune system. On the one hand, cytokines play a key role in modulating immune function by the CNS. In vivo experiment demonstrated that extracellular vesicles secreted by astrocytes in response to IL-1β easily penetrate the BBB and regulate leukocyte migration into the brain [125]. TNF-α administration to brain tissue increased the expression of CCL2 protein in the liver, stimulating the release of leukocytes into the peripheral circulation [126]. In patients after brain damage (brain tumor surgery), the number of leukocytes increased on the first day after surgery, compared to patients after minimally invasive surgery, such as aneurysm clipping [127]. In studies conducted by Sun Y et al. (2019) [128], IL-1β, IL-6, and CCL2 concentrations in the serum of patients with mild TBI were significantly higher than in healthy volunteers. Cytokine levels decreased 3 months after injury, but CCL2 levels remained high. Another study found elevated serum IL-1β, IL-10, IL-6, IL-8, IFN-α, and TNF-α levels in patients with TBI, even after 30 days of observation. Moreover, after treatment, levels of some of these cytokines (IL-6 and IL-8) remained higher than in the control group. Cytokine concentrations correlated with the severity of cognitive impairment as measured by the Montreal Cognitive Assessment Scale [129].

**Figure 2:**
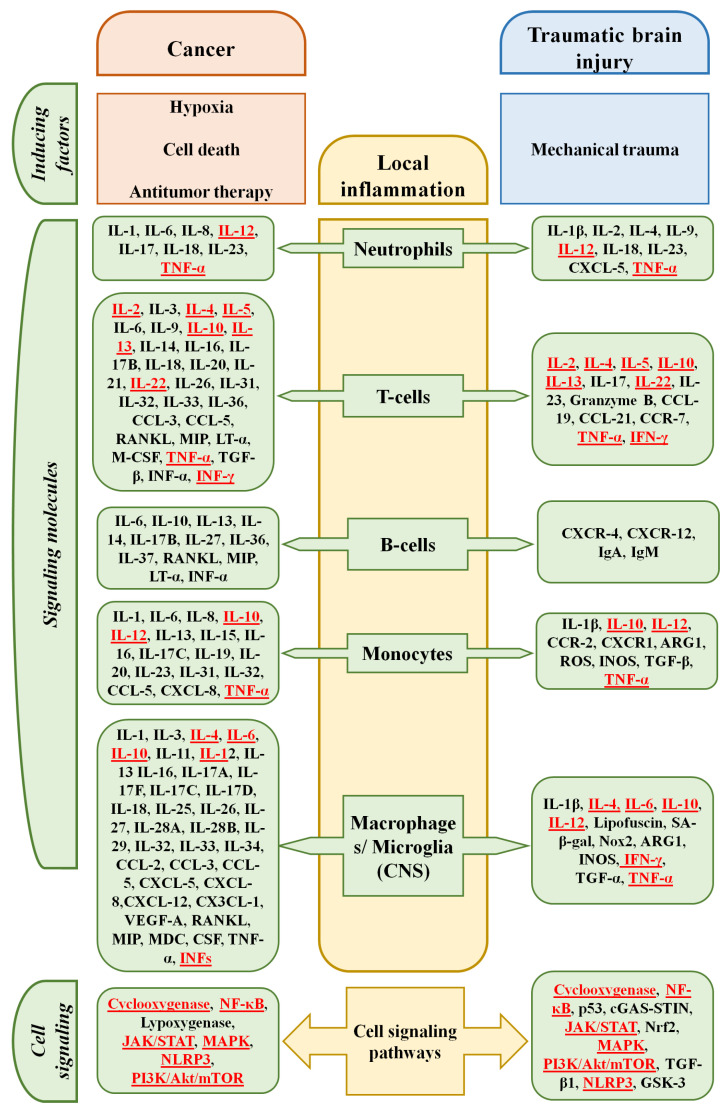
Key players in the development of local in cancer and traumatic brain injury [82,134]. CNS—central nervous system. Molecules that play a role in both cancer development and the formation of pathological changes in TBI are highlighted in red.

Systemic inflammation, in turn, modulates brain function. In this case, the cytokine targets are endothelial cells, pericytes, as well as glial cells (astro- and oligodendrocytes, microglia) and neurons [113,130]. In experiments, IFN-α reduced dopamine and serotonin levels in the animal brain. IL-1β and IFN-γ stimulated glutamine secretion by astrocytes [131]. IL-1β worsened the course of TBI, increasing the number of dead neurons and the area of damage [132]. Concurrently, splenectomy performed in rats immediately after severe TBI reduced animal mortality by reducing the severity of cerebral edema and decreasing the content of pro-inflammatory cytokines IL-1β, TNF-α, and IL-6 in blood serum and brain tissue [133]. The major parts of inflammation in cancer and traumatic brain injury are shown in Figure 2.

## 6. Methods for Reducing Inflammation and Reversal of T-Cell Exhaustion

### 6.1. Cancer

While acute inflammation is aimed at eliminating pathogens and the consequences of damaging factors, chronic inflammation is a factor of disease progression and adverse outcomes. Chronic inflammation is a component of the tumor microenvironment and contributes to the formation of an immunosuppressive environment in cancer, so its correction is essential both to improve the effectiveness of antitumor therapy and to prevent disease progression [135]. Nonsteroidal anti-inflammatory drugs (NSAIDs) exert their anti-inflammatory properties by inhibiting cyclooxygenase, an enzyme involved in prostaglandin synthesis. Other targets of NSAIDs’ action include transcription factors (NF-κB, AP-1, Sp1), AMPK/mTOR, PDPK-1 kinases, PPAR-γ, PPAR-δ, and RXRα nuclear receptors, which are involved in maintaining inflammation in cancer [135].

Drugs such as acetylsalicylic acid, nimesulide, and celecoxib have demonstrated potential antitumor effects in preclinical and clinical studies (Table 1). Despite the potential for NSAIDs to prevent and treat cancer, their widespread use is limited by side effects and the lack of sufficiently large clinical studies [136].

Statins, HMG-CoA reductase inhibitors, have long been established as lipid-lowering agents. However, their pleiotropic effects may be useful in modulating inflammation in malignancies. The anti-inflammatory effect of statins in cancer is associated with inhibition of the NLRP3 signaling pathway, reduced activation of the NF-κB pathway, decreased DAMP release, and modulation of the MAPK signaling pathway [152]. Clinical observations show some statin effectiveness in various types of cancer. However, further research into the effects of these medicines in oncological diseases is needed [153].

Glucocorticoids have powerful anti-inflammatory properties and can be used as additional drugs to reduce the side effects of the anti-cancer treatment [154]. However, the use of systemic glucocorticoids in cancer is limited due to their controversial effects on the immune system. They reduce pro-inflammatory cytokine production, suppress TLR-mediated signaling, and reduce the functional activity of T-cells [155]. Therefore, despite the improvement in survival rates of cancer patients when glucocorticoids and chemotherapy are administered in combination, their potential stimulating effect on the tumor process cannot be completely ruled out [154].

Immune checkpoint inhibitors are currently widely used to treat various types of cancer. Systematic reviews and original articles have detailed the anti-cancer effect of PD-1 blockers and their effects on exhausted T-cells [156,157,158]. In the development of the PD-1 blocker effects, their influence on the co-inhibitory interaction between dendritic cells and T-cells of the lymph nodes draining the tumor is important, Treg proliferation, differentiation of exhausted T-cell precursors into terminally exhausted T-cells [159,160,161,162,163,164].

Modifying the intracellular metabolism of immune cells may be a promising approach to enhancing the effectiveness of the antitumor response. Unlike genetic reprogramming, metabolic reprogramming of cells does not lead to changes in cellular identity [165].

Studies conducted by Verma V. et al. (2021) showed an increase in the antitumor effect of CD8^+^ cells upon inhibition of the MAPK/ERK signaling pathway [166]. Reprogramming with a mitogen-activated protein kinase 1/2 inhibitor (iMEK) and nivolumab, a human monoclonal antibody, also has a positive effect on T-cell cytotoxicity. Preclinical studies have shown that reprogrammed CD3^+^CD8^+^ T-cell therapy inhibits tumor growth and metastasis in Lewis lung carcinoma mouse model [167,168].

Other approaches to combating exhaustion include targeting co-inhibitory/co-stimulatory molecules. For example, preclinical studies of co-stimulatory receptor agonists CD28 and CD137 have shown their ability to enhance T-cell activation, improve mitochondrial function, and increase cytokine production [169,170]. The use of transcription factors and epigenetic modification to combat T-cell exhaustion is being considered, as well as cytokine therapy [171].

### 6.2. Traumatic Brain Injury

PD-1/PD-L1 blockade is a promising therapeutic option for various cancer types, including lung cancer, melanoma, colorectal cancer, and malignant tumors of the kidney, liver, and other organs. Meanwhile, their agonists are being explored as potential therapeutics for diseases of the nervous system. In a mouse model of ischemic stroke, PD-1/PD-L1 axis activation reduced mortality in the first 48 h after cerebral artery occlusion and moderated the severity of cerebral edema [172]. Administration of soluble PD-L1 to mice with cerebral vasospasm prevented infiltration of brain parenchyma by activated monocytes (PD-1^+^Ly6c^+^CCR2^+^) [173]. However, according to ClinicalTrials.com, no registered clinical trials are evaluating the efficacy of PD-L1 agonists in TBI or neurodegenerative diseases as of 1 November 2025.

Statins, nonsteroidal anti-inflammatory drugs, and glucocorticoids have been proposed as compounds for correcting neuroinflammation in brain diseases, including TBI. Phosphodiesterase inhibitors, TNF-alpha inhibitors, and IL-1 inhibitors are also useful for treating patients with TBI. The potential effects of these drugs are presented in Table 2 [174]. It should be noted that despite the large number of molecules with potential anti-inflammatory effects and their effectiveness in animal models of TBI, only a small number of them have shown their effect in patients with TBI and are undergoing clinical trials [175].

The involvement of the SNS in regulating immune cell function underlies the use of adrenergic agents in the treatment of TBI. Dexmedetomidine, an α2-adrenergic receptor agonist in the central nervous system, demonstrated a reduction in the proinflammatory cytokine production and the induction of M2 microglial polarization in rats with spinal cord injury due to increased PD-1 expression [176]. Dexmedetomidine reduced IL-1β, IL-6, and IL-8 production by mouse splenocytes, leading to decreased serum levels of these cytokines after TBI [177]. High dose of dexmedetomidine (200 mg/kg) inhibited microglial migration and reduced CD8^+^ T-cell numbers in the brain 72 h post-TBI [178]. The drug decreased the degree of damage to the BBB and suppressed the NF-κB activation in the cerebral cortex [179]. Administration of dexmedetomidine resulted in a significant decrease in the concentration of IL-1β, IL-6, IL-8, and TNF-α in the blood serum of patients with TBI [180].

Minocycline, a tetracycline antibiotic, has demonstrated some therapeutic potential in TBI. Its administration was associated with a reduction in brain infiltration by CD3^+^ T-cells and Ly6C^+^ monocytes and a decrease in the number of MCH II^+^ microglia [181]. In addition, the pleiotropic effects of minocycline included a decrease in the production of pro-inflammatory cytokines (IL-1β, IL-6, CCL8, CXCL4), inhibition of MAPK and NF-κB signaling pathways with a decrease in M1 microglial polarization, and activation of the TrkB/BDNF pathway, inducing M2 microglial polarization [182]. Despite the large number of studies evaluating the effectiveness of minocycline in animals with TBI, the ClinicalTrials list only two clinical studies. In moderate to severe TBI (NCT01058395), minocycline administration at 800 mg (loading dose) and 400 mg (maintenance dose) improved scores on the Disability Rating Scale [183]. Study NCT05826912 plans to evaluate the effects of several drugs, including minocycline, on functional (GOSE 2-Ways scale), cognitive impairments (Brief Test of Adult Cognition by Telephone, BTACT), and the severity of post-concussion syndrome (Rivermead Post Concussion Symptoms Questionnaire) in patients with TBI.

Other approaches are aimed at reducing secondary brain damage associated with the migration of immune cells, oxidative stress, etc. Fingolimod, a sphingosine-1-phosphate receptor modulator used to treat multiple sclerosis, reduced brain tissue infiltration by inflammatory cells (T-cells, NK-cells), microglia activation, increased the concentration of the anti-inflammatory cytokine IL-10, and the M2 to M1 ratio in C57BL/6 mice on day 3 post-TBI [184]. Glucocorticoids are known for their anti-inflammatory and immunosuppressive effects. However, their effect on neuroinflammation can be more complex. Administration of cortisol 1 h after lipopolysaccharide (LPS) reduced IL-1β and TNFα mRNA in the hippocampus. Administration of glucocorticoids before LPS injection had the opposite effect, increasing IL-6 production [185]. Study ISRCTN74459797, published in 2005, showed that glucocorticoid administration 48 h after TBI slightly increased the risk of death (25.7% vs. 22.3% in the placebo group) and death or disability (38.1% vs. 36.3%, respectively) in patients post-injury [186].

Although cyclosporine A has demonstrated neuroprotective properties in experimental TBI models, it has not shown an effect on neurological function in patients with TBI [187]. Conceivably, despite the potential therapeutic possibilities of using immunomodulatory agents such as glucocorticoids or cytostatics, the large number of serious side effects is the reason for the low interest in developing drugs based on them for the treatment of TBI and neuroinflammation.

Antibodies to pro-inflammatory cytokines are potential agents for TBI treatment. For example, anti-IL1β antibodies reduced microglial activation in animal models of TBI [188]. In clinical studies, the administration of a recombinant human interleukin-1 receptor antagonist was accompanied by an increase in the concentration of GM-CSF and IL-1β, which are associated with the activation of pro-inflammatory M1 microglia, while the concentration of IL-4, IL-10, and macrophage-derived chemoattractant, characteristic of M2, decreased [189].

Some authors point to biguanides as compounds capable of modulating the course of TBI. Study IRCT20180803040681N1 demonstrated that NLR returned to normal values more quickly in TBI patients treated with metformin, an oral hypoglycemic medication, than in untreated patients. Furthermore, metformin administration had a positive effect on serum GFAP concentration dynamics. This makes metformin a potential molecule for modulating both systemic inflammation and neuroinflammation in TBI [190]. Table 3 shows clinical trials with TBA and neuroinflammation as indication.

## 7. Discussion

Malignant neoplasms are among the four most common noncommunicable diseases [191]. TBI, despite a decreasing number of registered cases, makes a significant contribution to the overall structure of morbidity and mortality in people of different ages [192]. Although TBI and cancer represent pathologies with different etiologies and clinical courses, they are both characterized by immune system disorders and the development of local and systemic inflammation. In both cases, the PD-1/PD-L1 axis, as well as the SNS, is involved in the development of CD4^+^ and CD8^+^ T-cell exhaustion and are obligatory regulatory nodes. Additionally, other checkpoints, such as CTLA-4 and LAG-3, are involved in cancer, but their role in TBI remains poorly understood. This limits our understanding of immune post-TBI dysfunction and hinders the development of targeted therapeutic approaches.

In cancer, potential methods aimed at restoring antitumor immunity include immune checkpoint inhibitors, cytokines, and cell therapy. Approaches to regulating the tumor microenvironment and correcting inflammation, a key pathogenic factor, are being explored. However, the way to correct inflammation in TBI remains limited. The potential of NSAIDs, statins, and glucocorticoids in neuroinflammation is still being explored, and the development of methods to influence the cellular component of the immune response is hampered by a lack of a complete understanding of immune post-TBI regulation.

Thus, this review highlights significant gaps in the study of neuroimmune mechanisms in TBI and identifies areas for further research. Filling these gaps is critical for the discovery of new biomarkers and the development of therapeutic approaches aimed at correcting immune dysfunction after brain injury.

## Figures and Tables

**Table 1:** Antitumor effects of nonsteroidal anti-inflammatory drugs.

Nonsteroidal Anti-Inflammatory Drug	Effects	References
Acetylsalicylic acid	Chemoprevention of hepatocellular carcinoma in high-risk patients	[137]
	Inhibition of lung cancer cell growth (A549, H1299) in vitro	[138,139]
	Modulation of PD-L1 expression in vitro	
	Decreased survival of melanoma cells line A-375 in vitro	[140]
	Inhibition of breast cancer metastasis in vivo	[141]
	Enhancement of apoptosis of breast cancer cells in vitro	
	Inhibition of lung cancer metastasis	[142]
Nimesulide	Enhancement of TRAIL-induced apoptosis of Panc1 cells in vitro	[143]
	Inhibition of Panc1 cell proliferation in vitro	[144]
	Enhancement of apoptosis by Panc1 in vitro	
	Reduction in VEGF expression by tumor cells	
	Inhibition of proliferation of breast cancer cells (SK-BR-3, BT-474 and MDA-MB-453) in vitro	[145]
Celecoxib	Enhancement of the immune response in triple-negative breast cancer (in combination with paclitaxel)	[146]
	Inhibition of cervical cancer cell proliferation (HeLa, SiHa cell lines)	[147]
	Inhibition of epithelial–mesenchymal transition	[148]
	Stimulation of LAK-mediated lysis of lung cancer cells	[149]
	Decreased viability of Panc1 cells in vitro	[150]
	Inhibition of proliferation and stimulation of apoptosis in colon cancer cells (HCT-116)	[151]

**Table 2:** Drugs that affect neuroinflammation.

Group	Examples	Mechanisms of Influence on Neuroinflammation
Statins	Atorvastatin Lovastatin Simvastatin	Suppression of microglial activation
		Reduction in the proinflammatory cytokines production
		Suppression of TLR4, NF-κB, IL-1β, IL-6, TNFα, and ICAM-1 expression
Nonsteroidal anti-inflammatory drug	Indomethacin Ibuprofen Celecoxib Meloxicam Nimesulide	↓ IL-1β production Prostaglandin synthesis inhibition
TNF blockers	Etanercept	Reduce the level of IL-1β, IL-6, and TNFα
Phosphodiesterase inhibitors	Rolipram (only for experimental studies) Roflumilast	↓ IL-1β, IL-6, TNF-α, and NLRP3 level
Anti-IL-1 antibodies	Anakinra	↓ the level of IL-1β
		↑ the activity of antioxidant systems (superoxide dismutase, glutathione peroxidase)
Antibiotics	Minocycline	↓ IL-1β, IL-6, CCL8, CXCL4
		Inhibition MAPK and NF-κB signaling pathways
Immunosuppressants	Fingolimod	↓ severity of blood–brain barrier impairment
		↓ cerebral edema
		Modulation of immune cell functions
Others	N-Acetylcysteine	Reduction levels of IL-1β, TNFα, and IL-6
		↓ NF-κB expression
		↓ cerebral edema
		↓ severity of blood–brain barrier impairment
	Metformin	↓ IL-8 production
		↓ NF-κB pathway activity
		↓ microglial activation

↑ indicates upregulation (increase in expression or levels); ↓ indicates downregulation (decrease in expression or levels).

**Table 3:** Clinical trials targeting TBI and neuroinflammation registered as of October 2025 (ClinicalTrials, 2025).

	Traumatic Brain Injury	Neuroinflammation	Traumatic Brain Injury + Neuroinflammation
Stage			
Recruiting	385	64	6
Active	79	7	3
Completed	1117	65	7
Terminated	171	14	1
Phase			
I	108	26	4
II	232	33	9
III	129	5	1
IV	82	14	0

## Data Availability

Data are contained within the article.

## Abbreviations

The following abbreviations are used in this manuscript:

TBI	Traumatic brain injury
CRP	C-reactive protein
DAMPs	Damage-associated molecular pattern
MDSCs	Myeloid-derived suppressor cells
SNS	Sympathetic nervous system
CNS	The central nervous system
The BBB	The blood–brain barrier
NSAIDs	Nonsteroidal anti-inflammatory drugs
